# The effects of phosphorus limitation on carbon metabolism in diatoms

**DOI:** 10.1098/rstb.2016.0406

**Published:** 2017-07-17

**Authors:** Tore Brembu, Alice Mühlroth, Leila Alipanah, Atle M. Bones

**Affiliations:** Department of Biology, Norwegian University of Science and Technology, 7491 Trondheim, Norway

**Keywords:** carbon metabolism, diatom, phosphorus, proteome, transcriptome

## Abstract

Phosphorus is an essential element for life, serving as an integral component of nucleic acids, lipids and a diverse range of other metabolites. Concentrations of bioavailable phosphorus are low in many aquatic environments. Microalgae, including diatoms, apply physiological and molecular strategies such as phosphorus scavenging or recycling as well as adjusting cell growth in order to adapt to limiting phosphorus concentrations. Such strategies also involve adjustments of the carbon metabolism. Here, we review the effect of phosphorus limitation on carbon metabolism in diatoms. Two transcriptome studies are analysed in detail, supplemented by other transcriptome, proteome and metabolite data, to gain an overview of different pathways and their responses. Phosphorus, nitrogen and silicon limitation responses are compared, and similarities and differences discussed. We use the current knowledge to propose a suggestive model for the carbon flow in phosphorus-replete and phosphorus-limited diatom cells.

This article is part of the themed issue ‘The peculiar carbon metabolism in diatoms’.

## 1. Introduction

Phosphorus (P) is an essential element for all living organisms. It is a component of the backbone of DNA and RNA, and is also central in the transmission of chemical energy through adenosine triphosphate (ATP). Furthermore, P is also present in phospholipids, a major constituent of cell membranes. The main source of new bioavailable P to aquatic environments, in the form of inorganic phosphate (Pi, 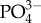), is weathering of continental rocks [1–3]. In addition, phytoplankton can use dissolved organic P (DOP), which is mainly available as phosphoesters, phosphonates and polyphosphates [4]. Pi is readily taken up by phytoplankton in the euphotic zone and incorporated in organic molecules. Plankton settling and subsequent remineralisation of P by bacteria result in the depletion of Pi in surface-ocean waters and increased Pi concentrations with depth [3,5]. DOP shows an opposite distribution, with highest concentrations near the surface [3,5].

Diatoms are unicellular, photosynthetic phytoplankton belonging to the heterokonts, and are found worldwide in freshwater and oceans. They are thought to be the ecologically most important group of eukaryotic phytoplankton, and contribute to at least 20% of the global CO_2_ assimilation as well as the biogeochemical cycling of important nutrients, such as carbon, nitrogen and silicon [6,7]. Diatoms arose through at least two endosymbiotic events [8,9]. Their complex evolutionary history has resulted in nuclear genomes that are a mix of genes of animal and plant/red algal origin [10,11]. In addition, a large number of diatom genes appear to be derived from horizontal gene transfer, mainly from bacteria [11–13].

Marine primary production is generally limited by the availability of nitrogen (N), iron (Fe), phosphorus and silicon (Si), of which the two former are considered to be the main limiting elements in most parts of the ocean [7,14]. P limitation has, however, been observed in the Mediterranean, the Gulf of Mexico and the Red Sea [5,7]. In other regions, such as the subtropical North Atlantic, N and P co-limitation appears to occur [7]. Under nutrient-replete conditions, such as seasonal or regional upwelling, diatoms grow and divide quickly, producing large blooms that dominate the phytoplankton communities [15,16]. In nutrient-poor, oligotrophic parts of the ocean, however, picoplankton and dinoflagellates are more dominant [17,18]. The ability to compete for limiting nutrients generally declines with increasing volume; due to their relatively large cell size, diatoms therefore are less competitive under low-nutrient conditions [19,20].

As a result of anthropogenic activities, the global availability of carbon and N is increasing without a parallel increase in P availability, thereby changing the stoichiometry of carbon and N relative to P [21]. Recently, Galbraith & Martiny [22] revealed a consistent relationship between particulate P:C ratios in phytoplankton and dissolved Pi concentrations. The close connection between P and carbon highlights the importance of understanding how carbon metabolism changes under low P conditions in phytoplankton including diatoms, both in current and future climate scenarios.

A number of reviews in the last few years have provided updated knowledge on the biogeochemical P cycle [2,3] as well as physiological and molecular mechanisms of P uptake, metabolism and storage in microalgae [5,23,24]. Here, we review the effect of P availability on carbon metabolism pathways in diatoms, using two transcriptome analyses as a basis, supported by other studies at transcriptome, proteome and metabolite levels. P, N and Si limitation studies are compared to identify general and nutrient-specific responses. Finally, a suggestive model for the changes in carbon flux in diatom cells under P limitation conditions is proposed.

## 2. Carbon metabolism responses to phosphorus limitation in diatoms

### (a) ‘Omics studies of P limitation responses—considerations and complications

With the advent of high-throughput techniques, researchers were able to take snapshots of parts and even the whole cellular inventory of mRNAs (‘transcriptomics’), proteins (‘proteomics’) or metabolites (‘metabolomics’) under different conditions. The publications of the *Thalassiosira pseudonana* [10] and *Phaeodactylum tricornutum* genomes [11] were crucial milestones, providing the first overviews of the cellular inventories of diatoms and facilitating the design of full-genome DNA microarrays. The development of RNA sequencing has removed the need for pre-existing knowledge of the genome sequence, as the transcriptomes can be assembled de novo [25].

A majority of the high-throughput studies of P limitation responses have been based on transcriptome profiling, using either full-genome DNA microarrays or RNA sequencing. The first ‘omics study of P limitation responses in diatoms was performed on *T. pseudonana*, and included both transcriptome and proteome analyses [26]. Subsequently, transcriptome or proteome analyses of P limitation have also been published for *P. tricornutum* [27–29], *Chaetoceros affinis* [30], *Skeletonema costatum* [31], and *Thalassiosira weissflogii* [32]. Table 1 summarizes the published profiling of transcriptome, proteome or metabolite responses to P limitation in diatoms.

**Table 1.** Summary of ‘omics experiments on P limitation in diatoms.

species	analysis	treatment	cell density (cells ml^−1^)	reference
*T. pseudonana*	transcriptomics, proteomics	100 h P depletion	6.0 × 10^6^ (+P), 4.0 × 10^6^ (−P)	[26]
*P. tricornutum*	transcriptomics	48 h P deprivation	8.3 × 10^6^ (+P), 7.7 × 10^6^ (−P)	[27]
*P. tricornutum*	transcriptomics	96 h and 192 h P depletion, 96 h P replenishment	4.8 × 10^6^ (+P), 1.3 × 10^6^ (96 h −P)	[28]
*P. tricornutum*	proteomics	48 h P deprivation	8.3 × 10^6^ (+P), 7.7 × 10^6^ (−P)	[29]
*T. weissflogii*	proteomics	144 h P depletion	1.5 × 10^6^ (+P), 6.5 × 10^5^ (−P)	[32]
*S. costatum*	transcriptomics	96 h P depletion, 4 h and 28 h resupplement with P or glucose-6-P	6.5 × 10^5^ (+P), 4.0 × 10^5^ (96 h −P)	[31]
*C. affinis*	transcriptomics	96 h and 120 h P deficiency	7.5 × 10^4^ (+P), 6 × 10^4^	[30]
			(96 h −P)	
*T. pseudonana*	phospholipid profiling	48 h P deprivation	1.5 × 10^6^ (+P), 1.4 × 10^6^ (−P)	[33]
*P. tricornutum*	glycerolipid profiling	13 d P depletion	n.a.	[34]

As with most types of stress responses, responses to P limitation can be divided into a stress-specific and a general secondary response. P-specific responses are directly related to increasing the availability of P, such as the induction of genes encoding P transporters or proteins scavenging P from DOP or internal P sources. The effects of P limitation or P starvation on most pathways of the carbon metabolism may be viewed as secondary. P-specific responses are strong and robust, and are consistently observed across photosynthetic organisms [23]. Responses of the carbon metabolism pathways, however, are variable between different studies, making general observations on these responses difficult. Some of these changes could be based on differences in tolerance to P limitation between diatom species or different strategies to adapt to this stress. However, differences in experimental design also appear to account for some of the discrepancies observed. As shown in table 1, there are major differences in the experimental set-ups of the different studies, e.g. with regard to the level of P withdrawal, the duration of the treatment and cell density. All these factors may influence carbon metabolism and the expression of its genes. Cell density affects light conditions, having a strong effect on photosynthesis and subsequently carbon metabolism. Thus, it is important to consider the experimental set-up when comparing results from different studies.

Carbon metabolism pathways in plant and algal cells are separated into different intracellular compartments, including the cytosol, chloroplasts, mitochondria, peroxisomes and the endoplasmic reticulum. This compartmentation enables the cell to adjust the carbon flux through regulation of transport of metabolites between the different compartments [35,36]. In order to obtain a correct overview of the carbon flow in diatom cells under different conditions, knowledge on the localization of the different enzymes is therefore important.

Three transcriptome datasets, from *T. pseudonana* [26] *P. tricornutum* [28] and *S. costatum* [31], respectively, were compared in order to gain an overview of the transcript level responses of the different carbon metabolism pathways to P limitation. *P. tricornutum* and *T. pseudonana* were chosen as the localization of the different isozymes are well predicted for these species [37,38]. The treatment was also similar in the three compared experiments (*P. tricornutum* and *S. costatum*, 96 h P depletion [28,31]; *T. pseudonana*, 100 h P depletion [26]). Expression data were only available for a few carbon metabolism pathways for *S. costatum*. The effect of P limitation on carbon metabolism pathways is summarized in table 2, and will be discussed in the next paragraphs.

**Table 2.** Comparison of transcriptional responses of carbon metabolism genes to P limitation in *T. pseudonana* [26], *P. tricornutum* [28] and *S. costatum* [31]. Mean log2 fold changes in the transcript levels of each pathway are indicated with the following symbols: +++>1.5; 1.5 >++>1.0; 1.0>+>0.5; 0.5<−<−0.5; −0.5 <÷<−1.0; −1.0 <÷÷<−1.5; −1.5 <÷÷÷. +/÷ indicates a mix of upregulated or downregulated genes in the pathway. NA, not assessed; CCM, carbon-concentrating mechanism; OPPP, oxidative pentose phosphate pathway; PLC/PLD, phospholipase C/D; TCA, tricarboxylic acid.

process/pathway	*T. pseudonana*, 100 h P limitation	*P. tricornutum* 96 h P limitation	*S. costatum* 96 h P limitation
CCM	−	−	NA
Calvin cycle	−	÷	NA
glycolysis, cytosol	++	+++	+++^a^
glycolysis, chloroplast	−	÷	+++^a^
glycolysis, mitochondria	−	+++	+++^a^
TCA cycle	−	+	NA
pyruvate metabolism	+/÷	+/÷	NA
cytosolic OPPP	+	++	NA
chrysolaminarin biosynthesis	+	−	NA
chrysolaminarin degradation	++	++	NA
fatty acid biosynthesis	÷	÷÷÷	NA
fatty acid β-oxidation	−	−	NA
Kennedy pathway	−	+/÷	NA
phospholipid degradation (PLC/PLD)	+	+	+++
galactolipid biosynthesis	−	−	NA
sulfolipid biosynthesis	++	+++	+++
betaine lipid biosynthesis	NA	+++	+++

^a^No information regarding subcellular localization.

### (b) The central carbon metabolism—CCMs, Calvin cycle, glycolysis, gluconeogenesis, TCA cycle and OPPP

Enzymes involved in CO_2_-concentrating mechanisms (CCMs), such as carbonic anhydrases and bicarbonate transporters, do not show a specific transcript level trend in *P. tricornutum* and *T. pseudonana* [26,28]. Calvin cycle-related genes show reduced expression in P-limited *P. tricornutum* cultures, but are unregulated in *T. pseudonana*. This could be related to reduced photosynthetic capacity under P limitation in *P. tricornutum*, which also has been shown for other diatom species [31,39–42]. Assuming that the P-limiting conditions were similar in both experiments, *T. pseudonana* might be less sensitive to P limitation. Whereas *P. tricornutum* stopped dividing after 48 h of P limitation, *T. pseudonana* grew for 124 h before cell division halted [26,28]. Triose phosphates produced by the Calvin cycle enter the plastidic glycolysis or are exported to the cytosol by triose phosphate transporters (TPTs); the specificities of diatom TPTs have not yet been characterized [43]. Glyceraldehyde-3-phosphate as well as 3-phosphoglycerate might be the most actively transported metabolites.

Glycolysis in diatoms has a complicated organization, as complete or partial glycolysis pathways have been predicted in three cellular compartments: the cytosol, chloroplast and mitochondria [37,38,44]. Furthermore, the diatom genomes sequenced to date all differ in the composition of the glycolysis pathway predicted to each compartment. A survey of genes involved in carbon partitioning metabolism in *T. pseudonana*, *P. tricornutum* and *Fragilariopsis cylindrus* revealed that several glycolytic enzymes exist as a variable number of isoenzymes, and that a subset of these isoenzymes are unique for one or two of the species [38]. Mitochondria in *T. pseudonana*, *P. tricornutum* and *F. cylindrus* are all predicted to contain the lower half of glycolysis, while the chloroplastic pathway lacks one or more glycolytic isozymes [37,38]. Finally, part of the chloroplastic glycolysis is shared with the Calvin cycle. All transcriptome and proteome studies on P limitation in diatoms reported increased levels of transcripts and proteins involved in glycolysis or gluconeogenesis [26–32]. However, when categorizing glycolysis isozymes based on their predicted localization, a more nuanced picture emerges. Transcripts of the cytosolic glycolysis pathway are generally upregulated in both *T. pseudonana*, *P. tricornutum* and *S. costatum* during P limitation (table 2). In *P. tricornutum*, mitochondrial glycolysis is also induced, whereas the chloroplastic pathway is moderately downregulated [28]. By contrast, mitochondrial and chloroplastic glycolysis are transcriptionally unregulated in *T. pseudonana* [26]*.* In plants, alternative reactions that enable cells to bypass ATP- or Pi-consuming reactions in glycolysis are believed to be activated upon P limitation, such as pyrophosphate-dependent phosphofructokinase or non-phosphorylating NADP-dependent glyceraldehyde-3-phosphate dehydrogenase [45]. A similar mechanism has also been suggested for diatoms [26]. While the transcriptome and proteome data do not clearly show any induction of such bypass reactions in diatoms, their role in P stress responses cannot be excluded.

Gluconeogenesis is basically a reversed glycolysis in which glucose is generated from pyruvate or oxaloacetate (OAA). The preferred compartment(s) for this pathway in diatoms is not known. OAA from the TCA cycle can be converted to phosphoenolpyruvate (PEP) by mitochondrial PEP carboxykinase (PEPCK); *PEPCK* expression in *P. tricornutum* is induced by P limitation [28]. *PEPCK* is unregulated in P-limited *T. pseudonana*, but transcript levels of cytosolic pyruvate phosphate dikinase (PPDK), which initiates gluconeogenesis from pyruvate, increases [26]. Both PEPCK and PPDK may take part in a C4 CCM [46]; whereas the existence of a C4 CCM in diatoms is still controversial, these enzymes could fulfil other roles besides gluconeogenesis.

Phosphofructokinase and pyruvate kinase catalyse the only non-reversible steps in glycolysis, and are therefore the main regulatory enzymes of this pathway. Guerrini *et al.* [47] compared the activities of glycolytic enzymes in P-replete and P-limited cultures of the diatom *Achnanthes brevipes*. Interestingly, the activities of phosphofructokinase and pyruvate kinase were reduced twofold and sevenfold respectively, suggesting that glycolysis is inhibited during P limitation in *A. brevipes*.

The TCA cycle is only moderately affected at the transcript level by P limitation in *P. tricornutum* and T*. pseudonana* [26,28]. We recently investigated responses to P limitation in *P. tricornutum*, combining transcriptome analyses with metabolite profiling (L Alipanah *et al*. 2017, personal communication). Most metabolites of glycolysis and the TCA cycle that were detected showed unchanged or reduced levels after 72 h of P limitation. One notable exception was citrate, which showed strongly increased levels. Citrate is a well-known inhibitor of phosphofructokinase [48] and has also been shown to activate fructose 1,6 bisphosphatase, which catalyses the reverse reaction [49]. Thus, P limitation appears to shift the carbon metabolism toward gluconeogenesis.

The oxidative pentose phosphate pathway (OPPP) in diatoms appears to be cytosolic, in contrast to plants, where most steps of the pathway take place in the chloroplast [37,50]. In both *T. pseudonana* and *P. tricornutum* this pathway is induced at the transcript level by P limitation [26,28,30]. The OPPP provides reducing power for biosynthetic processes in the form of NADPH, and may be induced to counteract lower NADPH production as a consequence of reduced photosynthesis during P limitation. NADPH is also needed to regenerate antioxidants for removal of reactive oxygen species generated as by-products of photosynthesis and respiration [51]. It may also act in catabolism of ribose-5-phosphate, a by-product of nucleic acid degradation, which is a P scavenging process common in P limitation responses. A recent flux balance analysis of primary metabolites in *P. tricornutum* [52] suggests that the OPPP is not the metabolically preferred pathway under optimal growth conditions, in line with the stress-induced expression of this pathway.

### (c) Polysaccharide metabolism

Chrysolaminarin, a polysaccharide consisting of a β-1,3-linked backbone with infrequent β-1,6-linked branches, is the main storage carbohydrate in diatoms [53,54]. Chrysolaminarin is likely synthesized from UDP-glucose, in continuation of the gluconeogenesis pathway. Inversely, degradation of chrysolaminarin by exo- and endo-1,3-β-glucanases or β-glucosidases will produce glucose, which enters glycolysis through the activity of glucokinase [37,55]. In P-limited *T. pseudonana* and *P. tricornutum*, chrysolaminarin degradation-related genes are more induced than chrysolaminarin biosynthesis-related genes, indicating a net breakdown of chrysolaminarin during P limitation. UDP-glucose pyrophosphorylase (UGPase), which converts glucose-6-phosphate (G6P) to UDP-glucose [56,57], increases both at transcript and protein level during P limitation [26,28]. UDP-glucose is substrate for synthesis of chrysolaminarin, but also other UDP-sugars that may be incorporated into complex polysaccharides, as will be discussed below.

Extracellular polymeric substances (EPS) are organic polymers that are produced and secreted by microalgae, including diatoms [58,59]. EPS are mainly composed of complex heteropolysaccharides (extracellular polysaccharides, ECPS) with a high degree of branching [60], as well as glycoproteins, and fulfil a number of roles, including the gliding capabilities of pennate diatoms, protection against grazing, cell attachment and water retention [58]. Nutrient limitation has been shown to induce production and accumulation of extracellular polysaccharides in a number of diatom species [47,60–65]. Generally, P limitation is a stronger inducer of ECPS production than N limitation, although examples of the opposite result also exist [65]. The highest EPS production rates are generally observed during the transition from exponential to stationary growth, which coincide with the depletion of P [60,64]. As cell division halts, protein and membrane lipid synthesis is strongly reduced. Although photosynthesis, and consequently Calvin cycle activity, is reduced in P-limited cells, the production of carbon skeletons still exceeds the requirements. This excess carbon can be stored as carbohydrate (chrysolaminarin), lipid (TAG), or excreted as EPS [66].

The monosaccharide composition of the ECPS differs between different diatom species, but the major constituents generally are glucose, galactose, fucose and mannose [58,59,67]. Upon P limitation, the ECPS monosaccharide composition as well as the degree of polymerization change [60,65]; these changes also appear to be species-specific [60,64]. Most of the enzymes known to be involved in biosynthesis of the different monosaccharides that constitute ECPS are present in diatoms [67]. Substrates for these pathways are generated either through gluconeogenesis or chrysolaminarin degradation. Underwood and co-authors [68] showed, using inhibitors and 14C-labelling, that ECPS production was partially dependent on chrysolaminarin degradation. In contrast to the monosaccharide biosynthesis pathways, no enzymes involved in the assembly of ECPS have yet been identified in microalgae. Glycosyltransferases, which catalyse the generation of glycosidic bonds from phosphor-activated sugar donors, are believed to be central enzymes in synthesis of EPS repeating units [59].

EPS can be used as a carbon source by heterotrophic bacteria [69]. Furthermore, EPS produced by microphytobenthic diatoms contains N and P that is regenerated during bacterial degradation [70]. Interestingly, EPS produced by P-limited cultures of *C. affinis* is degraded and used less efficiently by a natural bacterial assembly compared with control and N-limited cultures [71]. In another experiment, bacterial growth was reduced in exudates from P-limited cultures of the diatom *Cylindrotheca closterium* compared with exudates from control cultures [72]. This phenomenon might contribute to the accumulation of mucilage that has occurred in the Adriatic Sea, which has low levels of P [34].

### (d) Lipid metabolism

Genes encoding enzymes of fatty acid biosynthesis, which occurs in the chloroplast, are strongly suppressed in *P. tricornutum* by P limitation, but only moderately downregulated in *T. pseudonana*. This difference may be connected with the growth phase of the cultures. The P-limited *T. pseudonana* cultures had not yet ceased dividing at the time of harvesting and would still require new membrane lipids [26].

A hallmark of phytoplankton responses to low P levels is the substitution of phospholipids with non-phosphorus lipids. This property has been observed for both cyanobacteria and eukaryotic microalgae [73]. Profiling of the glycerolipidome of *P. tricornutum* identified the phospholipid phosphatidylglycerol (PG), the galactolipids monogalactosyldiacylglycerol (MGDG) and digalactosyldiacylglycerol (DGDG), and the sulfoquinovosyldiacylglycerol (SQDG) as dominant lipids in the plastidic membranes, and phosphatidylcholine (PC) and the betaine lipid diacylglyceryl-hydroxy-methyl-*N,N,N*-trimethyl-β-alanine (DGTA) as major lipids in the extraplastidic membranes [33]. P limitation of *P. tricornutum* resulted in complete loss of phospholipids and their replacement by non-P containing lipids, mainly SQDG and DGTA [33]. Similarly, P-starved *T. pseudonana* replaced PG with SQDG and PC with another betaine lipid, diacylglycerylcarboxyhydroxymethylcholine (DGCC) [73,74]. As part of the P limitation response in plants, PC and phosphatidylinositol (PI) are processed by phospholipases to release phosphocholine and phosphoinositol, respectively, as well as diacylglycerol (DAG) [75]. Whereas phospholipases with specificity towards PC (PLD) and PI (PLC) are present in diatoms, no homologs of PG-specific lipases or acyl hydrolases have been identified in *P. tricornutum* or *T. pseudonana*. In line with phospholipid degradation during P limitation, induced expression of PLCs and PLDs are observed in *P. tricornutum*, *T. pseudonana* and *S. costatum* [26,28,31]. Genes encoding SQDG biosynthesis enzymes are induced under P limitation in both *T. pseudonana, P. tricornutum* and *S. costatum*, as well as in other diatoms [26,28,31,32]. However, P limitation does not influence expression of the SQDG biosynthesis gene *SQD2* in *C. affinis* [30]. This may point to a higher P storage capacity in *C. affinis*, or a lower level of P limitation in this experiment. A gene encoding a putative DGTA synthesis enzyme has been identified in *P. tricornutum*; its expression is strongly induced by P limitation both in this diatom [28] and *S. costatum* [31]. DGCC synthesis has not yet been characterized and the *T. pseudonana* genome does not encode any enzyme with similarity to DGTA synthesis enzymes [26].

Fatty acids synthesized in the chloroplast may be further processed to generate membrane lipids, or stored as triacylglycerols (TAGs). TAG biosynthesis is located in the endoplasmic reticulum, where fatty acyl-CoA molecules are serially attached to glycerol-3-phosphate [76]. The enzymes of this pathway, also termed the Kennedy pathway, are unregulated at transcript level in *T. pseudonana* and show diverging responses in *P. tricornutum* under P limitation. However, several reports have shown that TAG levels increase when diatom cultures become P depleted [27,28,33,77,78]. DAG produced by phospholipid degradation can potentially feed into the lower part of TAG biosynthesis. The P limitation-induced expression of PLCs and PLDs observed in both *P. tricornutum* and *T. pseudonana* indicates that phospholipid degradation may contribute significantly to TAG accumulation during early stages of P limitation, until phospholipids become depleted.

## 3. Comparison of P responses with other nutrient responses in diatoms

As previously mentioned, Si and N are also essential nutrients for diatom growth. Si is required for synthesis of the cell wall, or frustule, of most diatoms (a notable exception is *P. tricornutum*, which is able to grow in the absence of Si [79]). However, Si starvation does not substantially affect other cellular processes as Si is not a component of other biomolecules [80]. A recent transcriptome analysis of Si starvation responses in *T. pseudonana* showed that genes encoding chloroplast-localized pathways related to photosynthesis and carbon metabolism, such as pigment biosynthesis, Calvin cycle, glycolysis and fatty acid biosynthesis, were co-expressed during the first 24 h of Si starvation [81]. The expression peak of these genes coincided with chloroplast division. Accumulation of cellular lipids in the form of TAGs was observed from 8–12 h of Si starvation, and was proposed to result from ongoing fatty acid biosynthesis as cellular requirements for membrane biosynthesis decreased due to cell division arrest.

N is required for biosynthesis of proteins, nucleic acids and chlorophyll. A number of studies have analysed N limitation responses in diatoms on the transcriptome [82–85], proteome [86–88] or metabolite [89–91] level.

The negative effect of N limitation on photosynthesis is observed across photosynthetic eukaryotes [92]. Comparison of photosynthesis during N and P limitation in *P. tricornutum* indicates that N limitation has a stronger effect, at least in this species [33,93,94]. Consequently, transcript levels of photosynthesis- and carbon fixation-related genes are downregulated [83–86]. Reduced expression of genes related to glycolysis and the OPPP is also observed in most studies; however, the putative localizations of the glycolysis gene products were not considered [82–84]. TCA cycle transcripts are induced in all studies, likely to provide carbon skeletons that are used to re-assimilate N generated by protein degradation. Fatty acid biosynthesis is strongly suppressed at the transcript level [82–85]. In contrast, a recent proteome study reported increased levels of fatty acid biosynthesis enzymes after 24 h of N limitation in *P. tricornutum* [88]. The discrepancy may be explained by post-transcriptional regulation of this pathway; alternatively, fatty acid biosynthesis may not yet be affected at this (relatively) early stage of N limitation. TAG accumulation is observed during N limitation in a wide range of microalgae [76]. Interestingly, most TAG biosynthesis genes in *P. tricornutum* are not induced by N limitation, with the exception of a type II diacylglycerol acyltransferase (DGAT2D) [84,85]; *DGAT2D* expression appears not to be affected by P limitation [28]. In parallel, Levitan *et al.* [84] observed reduced protein levels of DGAT2D, again indicating that post-transcriptional regulation might play a role. These results led the authors to suggest that the accumulation of TAGs during N stress is a consequence of carbon allocation rather than induction of lipid biosynthesis genes.

Abida *et al*. [33] compared the glycerolipid profile in N- and P-limited *P. tricornutum* cells. MGDG content decreased under both conditions. However, the substitution of phospholipids with non-P containing lipids seen in P-limited cells was not observed during N limitation. Hence, this response is likely specific for P limitation. Metabolite profiling has been performed on *T. pseudonana* cultures during early (24 h) N limitation [90]. Again, accumulation of TAGs was observed, as well as a 10-fold increase in citrate, suggesting that this is not necessarily a feature that is specific for P limitation. An extended metabolomic analysis of 13 diatom strains showed that citrate levels increased in only four of these during N limitation [91].

Bender *et al*. [83] compared N limitation responses at the transcriptome level in three diatoms: *T. pseudonana*, *F. cylindrus* and *Pseudonitzschia multiseries*. The overall response was similar in all three species, as described above. However, differences in gene copy number were found for several metabolic enzymes. The pennate *F. cylindrus* and *P. multiseries* have three orthologues of fructose 1,6 bisphosphatase not found in the centric *T. pseudonana*. Such additional orthologues may provide more flexibility, since they are less evolutionarily constrained with regard to expression and activity.

## 4. A speculative model for carbon flow in diatoms during P-replete and P-limited conditions

Based on the studies described above, a picture emerges where the carbon flow in diatoms fundamentally changes during P limitation. Since a flux balance analysis of primary metabolites has been done for *P. tricornutum* [52], this species forms the template for the model.

**Figure 1.**
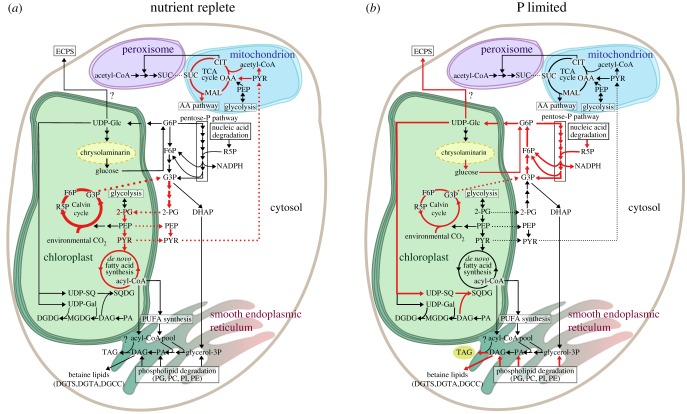
Speculative model on changes in carbon flux during P limitation in a diatom cell. (*a*) P-replete condition, based on a flux balance analysis in *P. tricornutum* [52]. (*b*) P-limiting condition. *P. tricornutum* is used as a template. Arrow thickness is correlated with relative carbon flux. Red arrows indicate the main carbon flow. Box, linear pathway; circle, cycle; dashed line, transportation between different compartments. Compartments: yellow dashed compartment, separate compartment for chrysolaminarin; blue, mitochondrion; purple, peroxisome; green, chloroplast. The question mark indicates an unknown pathway. Abbreviations: 2-PG, 2-phosphoglycerate; acyl-CoA, acyl-coenzyme A; acetyl-CoA, acetyl-coenzyme A; CIT, citrate; DAG, diacylglycerol; DGDG, digalactosyldiacylglycerol; DGCC, diacylglycerylcarboxyhydroxymethylcholine; DGTA, diaclyglycerylhydroxymethyltrimethylalanine; DGTS, diacylglyceroltrimethylhomoserine; DHAP, dihydroxyacetone phosphate; ECPS, extracellular polysaccharides; F6P, fructose-6-phosphate; G3P, glyceraldehyde-3-phosphate; G6P, glucose-6-phosphate; glycerol-3P, glycerol-3-phosphate; MAL, malate; MGDG, monogalactosyldiacylglycerol; OAA, oxaloacetate; PA, phosphatidic acid; PC, phosphatidylcholine; PE, phosphatidylethanolamine; PEP, phosphoenolpyruvate; PG, phosphatidylglycerol; PI, phosphatidylinositol; PUFA, polyunsaturated fatty acids; PYR, pyruvate; R5P, ribose-5-phosphate; SUC, succinate; SQDG, sulfoquinovosyl diacylglycerol; TAG, triacylglycerol; UDP, uridine diphosphate glucose; UDP-Gal, UDP-galactose; UDP-Glc, UDP-glucose; UDP-SQ, UDP-sulfoquinovose.

Figure 1*a* indicates the carbon flow under nutrient-replete, autotrophic growth conditions [52]. The high photosynthetic rate leads to high CO_2_ assimilation by the Calvin cycle. Cytosolic glycolysis is fed by triose phosphates from the Calvin cycle (glyceraldehyde-3-phosphate is shown in the figure). Since cytosolic enolase is lacking in *P. tricornutum*, cytosolic 2-phosphoglycerate will need to be reimported to plastids for conversion of phosphoenolpyruvate. Pyruvate produced by the chloroplast glycolysis (or imported from cytosol) can be processed to acetyl CoA, which enters the fatty acid biosynthesis pathway. An actively dividing cell needs to synthesize considerable amounts of phospholipids to generate cellular membranes for daughter cells. Pyruvate is also transported to the mitochondria where it enters the TCA cycle by conversion to oxaloacetate or via acetyl CoA. The TCA cycle is active under autotrophic conditions and produces ATP and carbon skeletons for amino acid biosynthesis to support cell growth.

Under P limitation (figure 1*b*), cell division eventually halts. Due to the reduced requirements for carbon skeletons for protein and phospholipid biosynthesis, more CO_2_ is fixed through the Calvin cycle than is consumed. In order to deal with the excess carbon, carbon flow is instead directed toward storage as TAG or excretion from the cell as ECPS. As part of the P scavenging strategy, phospholipids are degraded to release much-needed P and replaced with sulfolipids, galactolipids and betaine lipids. Phosphatidic acid and diacylglycerol produced through phospholipid degradation may also be converted to TAGs. Increased citrate levels lead to the inhibition of phosphofructokinase (and possibly activation of fructose bisphosphatase), thus switching at least the cytosolic glycolysis pathway towards gluconeogenesis. G6P may enter the OPPP, which is also fed with ribose 5-phosphate (R5P) from nucleic acid degradation. NADPH produced by the OPPP provides reducing power to the cell. G6P can also be converted to UDP-glucose, which is a substrate for the biosynthesis of chrysolaminarin, sulfolipids and galactolipids, as well as several of the sugars found in ECPS. ECPS is synthesized by uncharacterised glycotransferases and subsequently secreted.

## 5. Knowledge gaps and future directions

In the last few years a number of studies have provided a large amount of details on the response to P limitation in diatoms, especially on the transcript level. However, information on responses on the protein and metabolite levels is equally important to understand the effects of P limitation on carbon metabolism.

How does carbon flow through the central carbon metabolism during P limitation in diatoms? Quantification of the central carbon metabolism intermediates in P-limited cells combined with carbon flux analyses will likely provide valuable answers. Characterization of the translocators involved in transport of carbohydrates will also increase the understanding of how carbon flows between compartments. Activity analyses of the regulatory enzymes for glycolysis and gluconeogenesis during different stages of P depletion will provide information on the regulatory switch between glycolysis and gluconeogenesis during P limitation, and possible differences between the different compartments. Comparative analyses with other nutrient limitation treatments (N, Si, Fe) will bring more details regarding nutrient-specific and general limitation responses of carbon metabolism.

The ECPS biosynthesis pathway in diatoms is still completely unknown. What enzymes are involved in this process, and what is the significance of the changes in ECPS composition and polymerization degree during P limitation? Does the EPS have other roles in P limitation responses beyond acting as a carbon overflow outlet and protecting cells? How does EPS modulate ecological interactions between diatoms, grazers and heterotrophic bacteria during P limitation? From an applied point of view, reducing or blocking ECPS biosynthesis might redirect excess carbon flow toward TAG biosynthesis, thereby increasing TAG yield for biofuel production or other applications without affecting other parts of the central carbon metabolism.

Finally, a recent study by Alexander *et al.* [95] showed that two co-occurring diatom species (*Thalassiosira rotula* and *Skeletonema* spp.) display different capabilities in their N and P metabolism in the field, which may prevent direct competition for resources. It would be interesting to investigate whether species-specific properties of carbon metabolism in diatoms have a role in niche partitioning with regard to limiting P resources.

## Data accessibility

This article has no additional data.
